# Differential Effect of the Deletion of African Swine Fever Virus Virulence-Associated Genes in the Induction of Attenuation of the Highly Virulent Georgia Strain

**DOI:** 10.3390/v11070599

**Published:** 2019-07-02

**Authors:** Elizabeth Ramirez-Medina, Elizabeth Vuono, Vivian O’Donnell, Lauren G. Holinka, Ediane Silva, Ayushi Rai, Sarah Pruitt, Consuelo Carrillo, Douglas P. Gladue, Manuel V. Borca

**Affiliations:** 1 Agricultural Research Service, U.S. Department of Agriculture, Plum Island Animal Disease Center, Greenport, NY 11944, USA; 2 Animal and Plant Health Inspection, U.S. Department of Agriculture, Plum Island Animal Disease Center, Greenport, NY 11944, USA

**Keywords:** ASFV, ASF, African swine fever, porcine

## Abstract

African swine fever virus (ASFV) is the etiological agent of an often lethal disease of domestic pigs, African swine fever (ASF). The ASFV Georgia 2007 isolate (ASFV-G) is responsible for the current epidemic situation in Europe and Asia. Genetically modified ASFVs containing deletions of virulence-associated genes have produced attenuated phenotypes and induced protective immunity in swine. Here we describe the differential behavior of two viral genes, NL (DP71L) and UK (DP96R), both originally described as being involved in virus virulence. Deletion of either of these genes efficiently attenuated ASFV strain E70. We demonstrated that deletion of the UK gene from the ASFV-G genome did not decrease virulence when compared to the parental virus. Conversely, deletion of the NL gene produced a heterogeneous response, with early death in one of the animals and transient fever in the other animals. With this knowledge, we attempted to increase the safety profile of the previously reported experimental vaccine ASFV-GΔ9GL/ΔUK by deleting the NL gene. A triple gene-deletion virus was produced, ASFV-GΔ9GL/ΔNL/ΔUK. Although ASFV-GΔ9GL/ΔNL/ΔUK replicated in primary cell cultures of swine macrophages, it demonstrated a severe replication deficiency in pigs, failing to induce protection against challenge with parental ASFV-G.

## 1. Introduction

African swine fever (ASF) is a contagious viral disease of swine. The causative agent, ASF virus (ASFV), is a large enveloped virus containing a double-stranded (ds) DNA genome of approximately 190 kilobase pairs. ASFV shares aspects of genome structure and replication strategy with other large dsDNA viruses, including the Poxviridae, Iridoviridae, and Phycodnaviridae [1]. ASF causes a spectrum of disease that ranges from highly lethal to subclinical, depending on host characteristics and the virulence of circulating virus strains [2]. ASFV infection in domestic pigs is often fatal and is usually characterized by high fever, hemorrhages, ataxia, and severe depression.

Currently, the disease is endemic in more than twenty sub-Saharan African countries. In Europe, ASF is endemic on the island of Sardinia (Italy) and outbreaks of ASF have been recorded in the Caucasus region since 2007, affecting Georgia, Armenia, Azerbaijan, and Russia. The epidemic rapidly spread to Eastern Europe, and South and East Asia, including China. The virus causing this epidemic, ASFV Georgia 2007/1, is a highly virulent isolate that belongs to genotype II [3].

Currently, there is no vaccine available against ASF and disease outbreaks are usually controlled by quarantine and slaughter of affected and exposed herds. Pigs immunized with live attenuated ASFVs containing genetically engineered deletions of specific virulence-associated genes are protected when challenged with homologous parental viruses. Specifically, individual deletions of the UK (DP69R) gene [4], 23-NL (DP71L) gene [5], TK (A240L) gene [6], 9GL (B119L) gene [7,8], a group of 6–9 genes of the MGF360-530 [9], and DP148R [10] gene from the genomes of virulent ASFVs resulted in significant attenuation in swine. In addition, animals immunized with these modified recombinant viruses were protected from disease when challenged with their homologous parental viruses. So far, these observations are the only experimental evidence supporting rational development of attenuated virus strains.

Based on this information, gene deletions could be the methodological basis for rational development of live attenuated vaccines against different field isolates. However, most of the referenced gene deletions have only been tested in a limited number of virus isolates. The highly conserved 9GL gene has been deleted from three ASFV strains—Malawi, Pretoria, and Georgia 2010—resulting in attenuation with differential effectivity. Deletion of the NL gene completely attenuated the E70 strain [5] but did not affect virulence in the Malawi isolate [11]. Deletion of UK, DP148R, and two different versions of MGF360-530 have only been evaluated in one virus isolate [4,9,10,12].

Identification and characterization of virus genes associated with virulence are critical for vaccine development against emerging ASFVs. Here we describe the differential behavior of two viral genes, NL (DP71L) and UK (DP96R), originally described as determinants of viral virulence in the ASFV E70 strain [4,5], upon their individual deletion from the contemporary ASFV Georgia 2010 (ASFV-G) strain. Although the amino acid sequences of both virus genes are highly conserved between the E70 and Georgia strains, we show here that while the UK deletion (ASFV-G ΔUK) from the ASFV-G genome did not produce any decrease in virulence when compared with the parental virus, deletion of the NL gene produced partial attenuation of ASFV-G. We conclude that the differential effect of these two virus genes is dependent on other factors within the virus genome.

## 2. Materials and Methods

### 2.1. Cell Cultures and Viruses

Primary swine macrophage cell cultures were prepared from defibrinated swine blood as previously described [5]. Briefly, heparin-treated swine blood was incubated at 37 °C for 1 h to allow sedimentation of the erythrocyte fraction. Mononuclear leukocytes were separated by flotation over a Ficoll-Paque (Pharmacia, Piscataway, NJ, USA) density gradient (specific gravity, 1.079). The monocyte/macrophage cell fraction was cultured in plastic Primaria (Falcon; Becton Dickinson Labware, Franklin Lakes, NJ, USA) tissue culture flasks containing macrophage media, composed of RPMI 1640 Medium (Life Technologies, Grand Island, NY, USA) with 30% L929 supernatant and 20% fetal bovine serum (HI-FBS, Thermo Scientific, Waltham, MA, USA) for 48 h at 37 °C under 5% CO_2_. Adherent cells were detached from the plastic by using 10 mM EDTA in phosphate buffered saline (PBS) and were then reseeded into Primaria T25, 6- or 96-well dishes at a density of 5 × 10^6^ cells per mL for use in assays 24 h later.

ASFV Georgia (ASFV-G) was a field isolate kindly provided by Dr. Nino Vepkhvadze, from the Laboratory of the Ministry of Agriculture (LMA) in Tbilisi, Republic of Georgia.

Comparative growth curves between ASFV-G and recombinant viruses were performed in primary swine macrophage cell cultures. Preformed monolayers were prepared in 24-well plates and infected at a MOI (multiplicity of infection) of 0.1 (based on HAD_50_ (hemeadsorbing dose)previously determined in primary swine macrophage cell cultures). After 1 h of adsorption at 37 °C under 5% CO_2_, the inoculum was removed and the cells were rinsed two times with PBS. The monolayers were then rinsed with macrophage media and incubated for 2, 24, 48, 72, and 96 h at 37 °C under 5% CO_2_. At appropriate times postinfection (pi), the cells were frozen at ≤−70 °C and the thawed lysates were used to determine titers by HAD_50_/mL in primary swine macrophage cell cultures. All samples were run simultaneously to avoid inter-assay variability.

Virus titration was performed on primary swine macrophage cell cultures in 96-well plates. Virus dilutions and cultures were performed using macrophage medium. Presence of virus was assessed by hemadsorption (HA) and virus titers were calculated by the Reed and Muench method [13].

### 2.2. Construction of the Recombinant Viruses

Recombinant viruses ASFV-G-ΔNL and ASFV-G-ΔUK were generated by homologous recombination between the parental ASFV genome and the corresponding recombination transfer vector following infection and transfection of swine macrophage cell cultures [5,8]. All recombinant transfer vectors were obtained by DNA synthesis (Epoch Life Sciences, Sugar Land, TX, USA) containing a selection cassette flanked on both sides with LoxP sites consisting of mCherry under the ASFV p72 late gene promoter. Recombinant transfer vector p72mCherryΔNL contains flanking genomic regions of the NL gene mapping l kbp to the left of position 184,073 and 1 kbp right of position 184,232 of the NL gene. This construction allowed the C-terminal 54 nucleotides of DP71L to remain as to not interfere with the open reading frame (ORF) of MGF-360-18R on the reverse strand. This construction created a 160-nucleotide deletion in the NL (DP71L) ORF (amino acid residues 1–53). Similarly, recombinant transfer vector p72mCherryΔUK contains flanking genomic regions of the UK gene mapping 1 kbp to the left and 1 kbp right of the UK gene but leaving the last 34 nucleotides of UK intact without a start codon. This construction created a 256-nucleotide deletion in the UK (DP69R) ORF between positions 184,331 and 184,586.

Triple gene-deleted ASFV-G Δ9GL/ΔNL/ΔUK was produced using the previously developed virus ASFV-G 9GL [8] and the recombinant transfer vector p72mCherryΔNL/ΔUK. p72mCherryΔNL/ΔUK contains flanking genomic regions of the NL gene mapping 1 kbp to the left of position 184,073 and 184,586 kbp right of the UK gene, respectively. This construction allowed the C-terminal 54 nucleotides of DP71L to remain as to not interfere with the ORF of MGF-360-18R on the reverse strand and creates a 514-nucleotide deletion in the NL/UK ORFs (nucleic acid residues 184,073–184,586).

Macrophage cell cultures were infected with ASFV-G or ASFV-G 9GL and transfected with the corresponding recombinant transfer vector as described elsewhere [8]. Recombinant viruses were purified to homogeneity by successive rounds of limiting dilution purification.

### 2.3. Next-Generation Sequencing (NGS) of ASFV Genomes

ASFV DNA was extracted from infected cells and quantified as described earlier. Full-length sequence of the virus genome was performed as described previously [14] using an Illumina NextSeq sequencer.

### 2.4. Detection of ASFV-Specific Antibodies

Anti-ASFV antibodies in sera of infected animals were quantified using an in-house developed ELISA. Antigen preparation and ELISA procedures were exactly as described elsewhere [15]. Briefly, Vero cells were infected with an ASFV-G adapted to replicate in Vero cells [16] until cytopathic effect reached 100%. The infected cells were resuspended in water containing protease inhibitor (Roche, New York, NY, USA), followed by the addition of Tween 80 (G-Biosciences, St. Louis, MO, USA) and sodium deoxycholate (Sigma, St. Louis, MO, USA) to a final concentration of 1% (*v*/*v*). Uninfected Vero cells were treated in the same manner, and these antigens were stored at <−70 °C. Maxisorb ELISA plates (Nunc, St. Louis, MO, USA) were coated with 1 µg per well of either infected cell or uninfected cell antigen. The plates were blocked with phosphate-buffered saline containing 10% skim milk (Merck, Kenilworth, NJ, USA) and 5% normal goat serum (Sigma). Each swine serum was tested at multiple dilutions against both infected and uninfected cell antigen. ASFV-specific antibodies in the swine sera were detected by an anti-swine IgG-horseradish peroxidase conjugate (KPL, Gaithersburg, MD, USA) and SureBlue Reserve peroxidase substrate (KPL). Plates were read at OD_630_ in an ELx808 plate reader (BioTek, Shoreline, WA, USA). Swine sera were considered positive for ASFV-specific antibodies if the OD_630_ ratio of the reaction against infected cell antigen to uninfected cell antigen was higher than 2.2.

### 2.5. Animal Experiments

Animal experiments were performed under biosafety level 3AG conditions in the animal facilities at Plum Island Animal Disease Center (PIADC) following a protocol approved by the PIADC Institutional Animal Care and Use Committee of the US Departments of Agriculture and Homeland Security (protocol number 225.04-16-R, 09-07-16). ASFV-G- recombinant viruses were assessed for their virulence relative to the parental ASFV-G virus using 80–90-pound commercial breed female swine. Five pig per group were inoculated intramuscularly (IM) with 10^4^ HAD_50_ of recombinant viruses or ASFV-G. Clinical signs (anorexia, depression, fever, purple skin discoloration, staggering gait, diarrhea, and cough) and changes in body temperature were recorded daily throughout the experiment.

ASFV-G-Δ9GL/ΔNL/ΔUK was assessed for its protective effect at 28 days post-inoculation (dpi) by IM challenge with 10^2^ HAD_50_ of highly virulent parental ASFV-G. Clinical signs (as described above) and changes in body temperature were recorded daily throughout the experiment.

## 3. Results

### 3.1. ASFV NL (DP71L) and UK (DP69R) Genes Are Highly Conserved between E70 Isolate and Georgia 2010 Isolates

**Figure 1:**
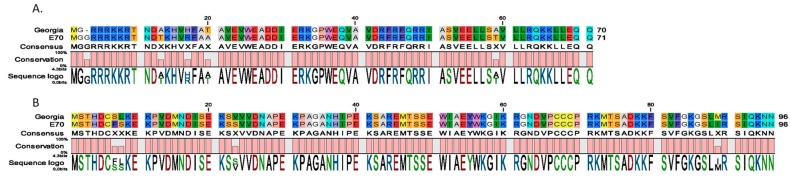
Alignment of the amino acid sequence obtained from full-length African swine fever virus (ASFV) proteins NL (DP71L) (**A**) and UK (DP96R) (**B**) from ASFV E70 and Georgia 2007 isolates.

Individual deletion of either NL (DP71L) or UK (DP69R) genes from the genome of the Spanish isolate E70 caused a drastic decrease in virus virulence when inoculated in swine under experimental conditions [4,5]. Interestingly, sequence comparison of the genes between the E70 and Georgia 2010 isolates reveals a high degree of conservancy. Amino acid identities of translational products of predicted open reading frames (ORFs) of ASFV Georgia 2010 (Assession# FR682468.1) and E70 genomes (Assession#: AF015671.1 and U73713) were compared using CLC Genomics Workbench 11.0 (https://www.qiagenbioinformatics.com/) and Basic Local Alignment Search Tool (BLAST) [17]. Between these isolates, amino acid identity for NL (DP71L) and UK (DP69R) genes are 94% and 96%, respectively (Figure 1). In addition, a total of 39 predicted ORFs are available for UK (DP69R) and it was observed that 28/39 of the predicted ORFs of the predicted proteins encoded by these ORFs retain a high percentage of identity (>90%); 7/39 have identities ranging between 80% and 90% and 4/39 are 70–80% identical. In the case of NL (DP71L), 39 predicted ORFs retained a high percentage of identity (>90%). The high degree of homology of these two genes between E70 and Georgia 2010 isolates would predict that their individual deletions in the Georgia 2010 genome would have similar decreased virus virulence phenotypes in swine infected with ASFV Georgia 2010.

### 3.2. Development of the Recombinant Deletion Mutant ASFVs

To assess the individual role of NL (DP71L) or UK (DP69R) genes in virulence during infection in swine, recombinant viruses were developed using ASFV-G as parental virus individually harboring deletion of either gene (ASFV-G-ΔNL and ASFV-G-ΔUK, respectively).

**Figure 2:**
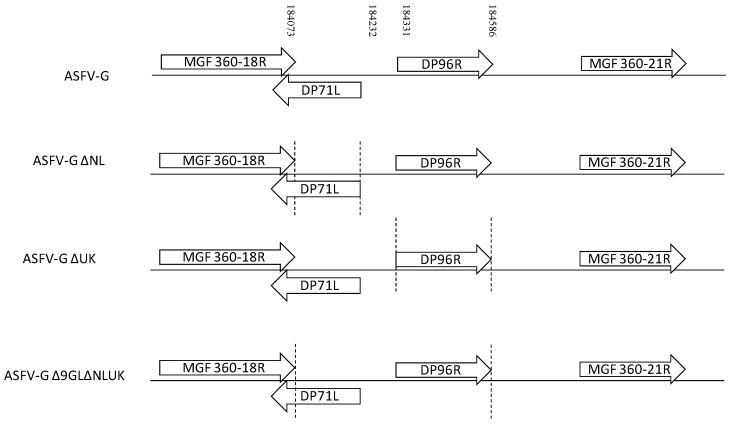
Schematic representation of the NL and UK gene regions deleted in recombinant viruses ASFV-G-ΔNL, ASFV-G-ΔUK, and ASFV-G-Δ9GL/ΔNL/ΔUK. The indicated region between the dotted lines is replaced with a p72mCherry cassette. Nucleotide positions indicating the boundaries of the deletion relative to the ASFV-G genome are indicated.

ASFV-GΔNL was constructed by genetic modification of the highly virulent ASFV Georgia 2010 isolate (ASFV-G) by homologous recombination with recombinant plasmid p72mCherryΔNL (Figure 2). A 156-bp region, encompassing amino acid residues 1–52, within the NL (DP71L) gene was deleted from ASFV-G virus and replaced with a gene cassette containing the mCherry gene under the control of the ASFV p72 late gene promoter (p72mCherry) by homologous recombination (see Material and Methods). Similarly, ASFV-GΔUK was constructed using ASFV-G as template and p72mCherryΔUK as recombinant plasmid. A 255-bp region, encompassing amino acid residues 1–85, within the UK gene (DP69R) was deleted and replaced with the p72mCherry cassette (Figure 2).

The recombinant viruses were obtained after successive purification events on monolayers of primary swine macrophage cell cultures. The virus populations obtained from the last round of purification were amplified in primary swine macrophage cell cultures to obtain a virus stock.

The accuracy of the genetic modification, the integrity of the genome of each of the recombinant viruses, and the purity of the stock virus populations were assessed by full genome sequencing of each of the recombinant deletion mutant viruses produced. ASFV-G-Δ9NL and ASFV-G-ΔUK full genome sequences were obtained using NGS on the Ion Torrent PGM™ and compared with the genome of parental ASFV-G. The analysis of the DNA sequence of two viruses confirms the accuracy of the designed deletions and their replacement in each case by insertion of 3295 nucleotides corresponding to the p72mCherry cassette. Besides the insertion of the cassette, no significant additional differences were observed between genomes. Therefore, recombinant deletion mutant viruses ASFV-G-ΔNL and ASFV-G-ΔUK did not accumulate any significant undesired mutations during the process of homologous recombination and consequent purification steps.

### 3.3. Replication of ASFV-G-ΔNL and ASFV-G-ΔUK in Primary Swine Macrophages

**Figure 3:**
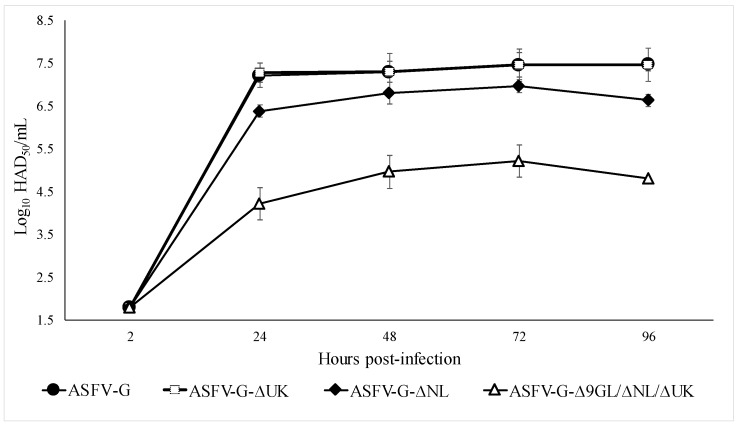
In vitro growth kinetics of recombinant viruses ASFV-G-ΔNL, ASFV-G-ΔUK, ASFV-G-Δ9GL/ΔNL/ΔUK, and parental ASFV-G. Primary swine macrophage cell cultures were infected (MOI = 0.1) with either recombinant virus or parental ASFV-G viruses. Virus yield was estimated at indicated times postinfection by titration in primary swine macrophage cell cultures. Data represent means and standard deviations from three independent experiments. Sensitivity of virus detection: >1.8 HAD_50_/mL.

In vitro growth characteristics of ASFV-G-ΔNL and ASFV-G-ΔUK were evaluated in primary swine macrophage cell cultures, the primary cell targeted by ASFV during infection in swine, and compared relative to parental ASFV-G in multistep growth curves (Figure 3). Cell cultures were infected at a MOI of 0.1 and samples were collected at 2, 24, 48, 72, and 96 h pi (hpi). Results demonstrate that ASFV-G-ΔUK displayed a growth kinetic significantly similar to parental ASFV-G virus. Depending on the time point and MOI utilized to infect macrophages, the recombinant virus exhibited similar titers relative to the parental virus. In the case of ASFV-G-ΔNL, the virus exhibits slightly decreased replication. Titers are approximately 10-fold lower than those of either ASFV-G or recombinant ASFV-G-ΔUK, however, there was no statistical difference between the three growth curves, except at 96 hpi (*p*-value = 0.003) where ASFV-G-ΔNL was lower. Results obtained with deletion of either NL [5] or UK genes [4] in the context of ASFV E70 did not affect the ability of either virus to replicate in vitro in primary swine macrophage cell cultures. Therefore, while deletion of the UK gene seems to not affect virus replication in either E70 or Georgia isolates, deletion of the NL gene in the Georgia isolate appears to have a slightly detrimental effect on virus replication in macrophages when compared with the effect of the homologous gene in the E70 isolate. 

### 3.4. Assessment of Either NL (DP71L) or UK (DP69R) Gene Deletion in ASFV-G Virulence in Swine

As already discussed, individual deletion of either NL (DP71L) or UK (DP69R) genes from the genome of the Spanish isolate E70 caused a drastic decrease in virus virulence when inoculated in swine under experimental conditions [4,5]. In those reports, it was observed that IM inoculation of pigs with the recombinant deletion mutant at doses as high as 10^4^ HAD_50_ only induced a transient rise in body temperature. Consequently, we decided to assess if similar gene deletions would similarly affect virulence of the highly virulent ASFV-G isolate. ASFV-G-ΔNL and ASFV-G-ΔUK viruses were experimentally inoculated in domestic swine and their virulence compared to that of their parental virulent virus ASFV-G.

Pigs inoculated via IM with 10^4^ HAD_50_ of ASFV-G exhibited increased body temperature (>104 °F) by 4–5 dpi. Pigs developed severe clinical signs associated with the disease including anorexia, depression, purple skin discoloration, staggering gait, and diarrhea. Signs of the disease aggravated rapidly over time and animals either died or were euthanized in extremis by 5–6 dpi (Table 1).

**Figure 4:**
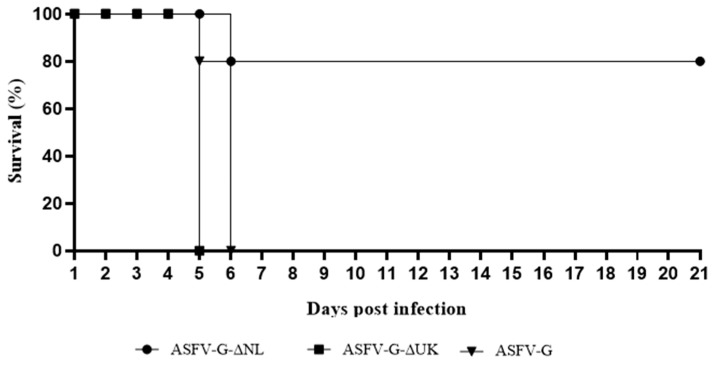
Evolution of mortality in animals infected with 10^4^ HAD_50_ of recombinant viruses ASFV-G-ΔNL, ASFV-G-ΔUK, or parental ASFV-G. Animals were monitored for an observational period of 21 days postinfection.

**Figure 5:**
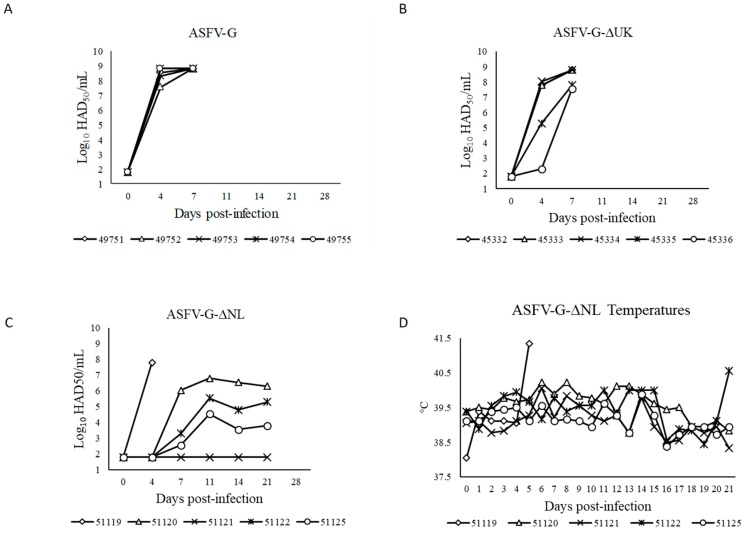
Virus titers in blood samples obtained from pigs infected with either 10^4^ HAD_50_ of parental ASFV-G (**A**), recombinant viruses ASFV-G-ΔUK (**B**), and ASFV-G-ΔNL (**C**). Values are expressed as log_10_ HAD_50_/mL. Sensitivity of virus detection: ≥10^1.8^ HAD_50_/mL. (**D**) Evolution of body temperature in pigs inoculated with ASFV-G-ΔNL.

Animals inoculated IM with 10^4^ HAD_50_ of ASFV-G-Δ9UK developed a clinical disease comparable in severity to that observed in animals infected with parental virus, ASFV-G. Pigs presented a short period of fever starting by 3–4 dpi, with animals dying or euthanized in extremis by 5 dpi. Interestingly, animals IM inoculated with 10^4^ HAD_50_ of ASFV-G-Δ9NL presented a heterogeneous behavior. One animal suddenly died at 5 dpi, presenting at necropsy with clinical lesions compatible with hyperacute disease. The four remaining animals remained clinically normal for 21 days, developing a delayed and subclinical disease when compared to that observed in animals infected with parental virus. Pigs presented a short period of fever starting at 4 dpi, followed by a recurrent period of transient rise in body temperature throughout the observational period (Table 1, Figure 4 and Figure 5D).

Viremia in experimentally inoculated animals was quantified at different days post-inoculation by HA (Figure 5A–C). As expected, animals inoculated with virulent parental ASFV-G had very high virus titers in blood until the day of their death. Similarly, animals inoculated with either ASFV-G-ΔUK had comparable virus titers in blood when compared with those of the ASFV-G-inoculated animals with just a small decrease in average titers by 4 dpi. In accordance with clinical presentation, in the group infected with ASFV-G-ΔNL the animal dead at 5 dpi presented with high viremia titers while the remaining four animals, which survived the infection, presented with heterogeneous behavior. One of them had constant relatively high viremia titers (around 10^6.36^ HAD_50_/_mL_, SD of 0.24) beginning at 7 dpi until the end of the observational period. A second animal presented with similar kinetics, averaging titers of 10^4.99^ HAD_50_/mL, SD of 0.55, while a third animal presented with average titers of 10^3.61^ HAD_50_/mL, SD of 0.82. These three animals all had recurrent transient increases in body temperature. Finally, a fourth animal did not present any detectable viremia at any of the time points tested. Consequently, viremia values closely correlate with the severity and kinetics of the presentation of clinical disease.

Surprisingly, deletion of UK does not alter virulence of the ASFV-G isolate, and deletion of the NL gene does not completely attenuate the virus. Considering the high homology between NL and UK genes between E70 and Georgia 2010 isolates, these results strongly stress the importance of genetic background when determining the role of these two genes in ASFV virulence.

### 3.5. Inclusion of Deletion of NL Gene in the Vaccine Candidate ASFV-G-Δ9GL/ΔUK Affects Its Protective Effect against Challenge with Virulent Parental Virus

Since deletion of NL induced a significant decrease in ASFV-G virulence it was tempting to assess if inclusion of the NL deletion would increase safety of the vaccine candidate ASFV-G-Δ9GL/ΔUK [18]. Therefore, a novel recombinant virus ASFV-G-Δ9GL/ΔNL/ΔUK was developed by deleting the NL gene from ASFV-G-Δ9GL/ΔUK genome. The strategy for developing ASFV-G-Δ9GL/ΔNL/ΔUK was similar to the one described to obtain ASFV-G-ΔNL/ΔUK (using p72mCherryΔNL/ΔUK as recombinant plasmid) but with ASFV-G-Δ9GL [8] as parental virus. Analysis of the ASFV-G-Δ9GL/ΔNL/ΔUK genome by NGS revealed absence of undesirable mutations, with the expected insertion of the p72mCherry cassette replacing the NL and UK genes the only significant genomic modification when compared with parental ASFV-G-Δ9GL.

The in vitro growth ability of ASFV-G-Δ9GL/ΔNL/ΔUK in swine macrophages was assessed and compared with that of the other recombinant viruses and the parental ASFV-G in a multistep growth curve experiment performed in primary swine macrophage cell cultures. Results demonstrate that ASFV-G-Δ9GL/ΔNL/ΔUK displayed a growth kinetic approximately 100-fold less when compared with ASFV-G-Δ9GL/ΔUK [18] and 1000-fold less when compared with the parental ASFV-G virus (Figure 3).

**Figure 6:**
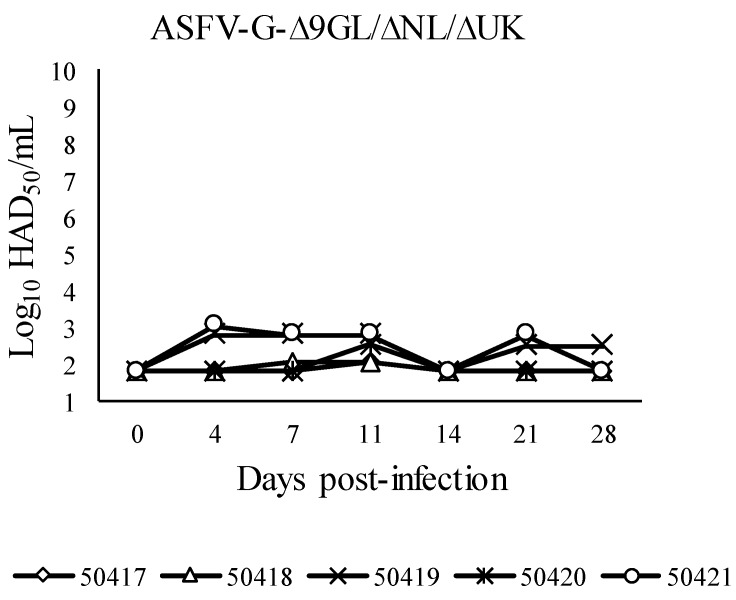
Virus titers in blood samples obtained from pigs infected with 10^4^ HAD_50_ of ASFV-G-Δ9GL/ΔNL/ΔUK. Values are expressed as log_10_ HAD_50_/mL. Sensitivity of virus detection: ≥10^1.8^ HAD_50_/mL.

To assess the consequence of the deletion of the NL gene in the protective effect of ASFV-G-Δ9GL/ΔUK, a group of 5 animals were IM inoculated with 10^4^ HAD_50_ and challenged 28 days later with 10^2^ HAD_50_ of virulent ASFV-G. As expected, all inoculated animals remained clinically normal until the day of challenge. Viremia kinetics showed a very low level of replication of ASFV-GΔ9GL/ΔNL/ΔUK (Figure 6), a differential fact when compared with those previously described for ASFV-G-Δ9GL [8] or ASFV-G-Δ9GL/ΔUK [18]. Consequently, specific ASFV antibody response elicited by ASFV-G-Δ9GL/ΔNL/ΔUK infection was very low (data not shown), much decreased compared to that induced by ASFV-G-Δ9GL/ΔUK [18].

After challenge, all ASFV-G-Δ9GL/ΔNL/ΔUK infected animals developed a clinical disease indistinguishable in kinetics and severity from that observed in challenged control animals. Animals in both groups presented with disease 4 dpi and were euthanized by 6 dpi (Table 2). Concomitantly, no differences were either detected in the kinetics or magnitude of viremias between mock-infected controls and the ASFV-G-Δ9GL/ΔNL/ΔUK infected pigs. These results demonstrated that deletion of the NL gene decreased the ability of the ASFV-G-Δ9GL/ΔUK vaccine candidate to replicate both in cell cultures and in vivo and to induce protection against challenge with parental virulent virus. It is possible that the low replication of ASFV-G-Δ9GL/ΔNL/ΔUK in pigs is responsible for its poor protective effect against challenge with virulent virus.

## 4. Discussion

No vaccines are available to prevent ASFV infection. Only live attenuated virus strains have been useful in protecting pigs against challenge with homologous virulent isolates [4,5,6,7,8,9,10,12,19]. These attenuated viruses have been regularly produced by sequential passages in cell cultures and, more recently, by genetic manipulation. Attenuated viruses obtained by genetic manipulation typically involve the deletion of specific genes associated with virus virulence. To date, independent deletion of only six different genes/group of genes has been shown to attenuate virulent ASFV. Independent deletions of the NL (DP71L) [5] or the UK (DP69R) [4] genes in ASFV E70 isolate, deletion of the TK (A240L) gene in ASFV adapted to Vero cells using Malawi Lil-20/1, Haiti [6], and Georgia isolates [20], deletion of the 9GL (B119L) gene from Malawi Lil-20/1 [7], Pretoriuskop/96/4 [15], and Georgia [8] isolates, deletion of six or nine genes of the MGF360/505 [9] of Georgia and Benin [12] isolates, respectively, and deletion of DP148R gene in Benin isolate [10] rendered recombinant deletion mutant viruses with significantly reduced virulence in swine. In all these cases, animals inoculated with each of these genetically modified viruses survived the infection and became protected against ASFV when challenged with the corresponding virulent parental virus (homologous challenge) [4,5,7,8,10,12,18]. These findings suggest that development of attenuated ASFV recombinant viruses by genetic manipulations of target genes is an effective approach for vaccine development. Cross-protection among different ASFV isolates is a quite rare event requiring the development of isolate-specific vaccines. This issue obligatorily raises the question of the reproducibility of the attenuating effect of a particular gene deletion in different virus isolates. In this regard, not many studies have been specifically focused on this important subject.

Deletion of the TK (A240L) gene, a highly conserved gene among all ASFV isolates, has been introduced into the genome of the pathogenic Vero cell-adapted Malawi Lil-20/1, Haiti H811, and Georgia viruses acquiring similar levels of attenuation, although they differed in their effect as potential vaccine virus [6,20].

The 9GL (B119L) gene is highly conserved among ASFV isolates sequenced thus far. Deletion of the gene from virulent Malawi Lil-20/1 [5] and Pretoriuskop/96/4 [15] effectively reduced virulence in swine and induced protection making 9GL, a candidate target gene for modification to produce attenuated live vaccines. However, more recently it was shown that 9GL deletion in the Georgia isolate does not have the same effect in terms of attenuation since only when administrated at a low dose to swine it was possible to observe a significant reduction in virus virulence of a Georgia isolate having 9GL deleted [8].

The NL gene has been shown to differentially behave in different virus isolates. Interestingly, the NL gene exists in two different forms: a long (184 amino acids) form, found almost exclusively in Malawi Lil-20/1 isolate, and a short form (70–72 amino acids), present in most of the ASFV isolates sequenced to date [5,11]. Deletion of this gene in the ASFV E70 isolate (short form) rendered an attenuated virus [5], while its deletion in ASFV Malawi Lil-20/1 (long form) did not result in attenuation of the virus [11]. The NL proteins encoded by E70 and Malawi Lil-20/1 differ significantly and that may explain the phenotypic differences observed in swine inoculated with the respective deletion mutant viruses. However, the short form of NL gene and the UK gene are quite conserved across all ASFV isolates. Protein identity matrices indicate that the NL and UK proteins are highly similar among ASFV isolates where E70 and Georgia share over 94% and 96% amino acid identity, respectively, making it unlikely that ASFV attenuation relies solely on protein divergence. Since the observed phenotypes are most likely mediated by the effect of multiple genes [4,5,7,8,9,10,12], the evidence accumulated so far makes it difficult to speculate what is indeed the spectrum of genes mediating virulence in the ASFV Georgia 2007 isolate. It is possible that the number or function of additional virulence-associated genes among different ASFV strains may alter the intrinsic effect of NL and UK genes on the general balance of the virulence in a particular virus strain. Therefore, it remains to be determined why the deletion of NL or UK, both genes independently associated with virus virulence in the E70 isolate, does not drastically alter virulence of ASFV-G. 

Data presented here indicate that the UK gene by itself is not conclusively required for ASFV Georgia isolate virulence, although it was shown that increased attenuation to the ASFV-G-Δ9GL vaccine candidate [18]. Similarly, the importance of NL in virus virulence in the ASFV-G isolate does not completely resemble that shown in ASFV E70 suggesting that other virulence-associated genes may be involved in the process of virulence in the Georgia isolate. As observed with deletions of the 9GL gene in Malawi Lil-20/1 [7], Pretoriuskop/96/4 [15], and Georgia [8] isolates leading to different phenotypes, deletions of NL and UK genes have produced similar outcomes, suggesting that ASFV virulence is the result of a multigene effect. These results suggest that the differential phenotypic effect observed after deleting virulence-associated genes from two different ASFV genomes is dependent on the genomic background of the viral isolate.

Supporting this hypothesis is the fact that attenuation of ASFV E70 isolate by deleting NL gene have been shown to be reverted by insertion of a group of MGF360/530 from ASFV Malawi isolate [21]. This result indicated that NL gene deletion was effective in inducing attenuation in ASFV E70 isolate because of the natural absence of a group of MGF genes which were later shown to be critical in virus virulence [9,12].

Therefore, despite high amino acid identity of the translated products of the UK and NL genes from E70 and Georgia viral isolates, other genetic factors must influence ASFV virulence. These genetic determinants of virulence require further characterization, likely on an isolate-by-isolate basis. This work indicates that rational development of novel ASFV vaccines requires caution, avoiding direct extrapolation of results obtained by specific gene deletions obtained in a particular virus strain to novel field virus isolates.

## Figures and Tables

**Table 1:** Swine survival and fever response in animals infected with mutant viruses ASFV-G-ΔNL or ASFV-G-ΔUK compared with those infected with parental ASFV-G.

			Fever		
Treatment ^1^	No. of Survivors/Total	Mean Time to Death (Days ± SD)	No. of Days to Onset (Days ± SD)	Duration No. of Days (Days ± SD)	Maximum Daily Temp (°F ± SD)
ASFV-G	0/5	5.8 (0.45)	5 (0.71)	1 (0.71)	106.62 (0.77)
ASFV-G-ΔNL	4/5	4 ^2^	6.7 (0.58)	1.67 (0.58) ^3^	103.68 (1.59)
ASFV-G-ΔUK	0/5	5 (0)	4 (1)	1 (1)	106.64 (0.32)

^1^ All animals were IM inoculated with 10^4^ HAD_50_ of the indicated virus. Animals were observed for 21 days after inoculation. ^2^ This value corresponds to the only animal euthanized in this group. ^3^ After initial peak of body temperature, there were intermittent and transient peaks in the surviving animals throughout the observational period (Figure 5D).

**Table 2:** Survival and fever response in animals infected with ASFV-G-Δ9GL/ΔNL/ΔUK and challenged 28 days later with 10^2^ HAD_50_ of parental ASFV-G.

			Fever		
Treatment	No. of Survivors/Total	Mean Time to Death (Days ± SD)	No. of Days to Onset (Days ± SD)	Duration No. of Days (Days ± SD)	Maximum Daily Temp (°F ± SD)
Mock vaccinated	0/5	6.8 (0.45)	4.2 (0.45)	1.8 (0.45)	104.34 (0.4)
ASFV-G-Δ9GL/ΔNL/ΔUK ^1^	0/5	6 (0)	4 (0)	2 (0)	105.8 (0.54)

^1^ All animals were IM vaccinated with 10^4^ HAD_50_.

## References

[1] Tu S.L., Staheli J.P., McClay C., McLeod K., Rose T.M., Upton C. (2018). Base-by-base version 3: New comparative tools for large virus genomes. Viruses.

[2] Costard S., Wieland B., de Glanville W., Jori F., Rowlands R., Vosloo W., Roger F., Pfeiffer D.U., Dixon L.K. (2009). African swine fever: How can global spread be prevented?. Philos. Trans. R. Soc. Lond. Ser. B Biol. Sci..

[3] Tulman E.R., Delhon G.A., Ku B.K., Rock D.L. (2009). African swine fever virus In Lesser Known Large Dsdna Viruses.

[4] Zsak L., Caler E., Lu Z., Kutish G.F., Neilan J.G., Rock D.L. (1998). A nonessential African swine fever virus gene UK is a significant virulence determinant in domestic swine. J. Virol..

[5] Zsak L., Lu Z., Kutish G.F., Neilan J.G., Rock D.L. (1996). An African swine fever virus virulence-associated gene nl-s with similarity to the herpes simplex virus icp34.5 gene. J. Virol..

[6] Moore D.M., Zsak L., Neilan J.G., Lu Z., Rock D.L. (1998). The African swine fever virus thymidine kinase gene is required for efficient replication in swine macrophages and for virulence in swine. J. Virol..

[7] Lewis T., Zsak L., Burrage T.G., Lu Z., Kutish G.F., Neilan J.G., Rock D.L. (2000). An African swine fever virus *ERV1-ALR* homologue, *9GL*, affects virion maturation and viral growth in macrophages and viral virulence in swine. J. Virol..

[8] O’Donnell V., Holinka L.G., Krug P.W., Gladue D.P., Carlson J., Sanford B., Alfano M., Kramer E., Lu Z., Arzt J. (2015). African swine fever virus Georgia 2007 with a deletion of virulence-associated gene *9GL* (b119l), when administered at low doses, leads to virus attenuation in swine and induces an effective protection against homologous challenge. J. Virol..

[9] O’Donnell V., Holinka L.G., Gladue D.P., Sanford B., Krug P.W., Lu X., Arzt J., Reese B., Carrillo C., Risatti G.R. (2015). African swine fever virus Georgia isolate harboring deletions of MGF360 and MGF505 genes is attenuated in swine and confers protection against challenge with virulent parental virus. J. Virol..

[10] Reis A.L., Goatley L.C., Jabbar T., Sanchez-Cordon P.J., Netherton C.L., Chapman D.A.G., Dixon L.K. (2017). Deletion of the African swine fever virus gene DP148R does not reduce virus replication in culture but reduces virus virulence in pigs and induces high levels of protection against challenge. J. Virol..

[11] Afonso C.L., Zsak L., Carrillo C., Borca M.V., Rock D.L. (1998). African swine fever virus NL gene is not required for virus virulence. J. Gen. Virol..

[12] Sanchez-Cordon P.J., Jabbar T., Berrezaie M., Chapman D., Reis A., Sastre P., Rueda P., Goatley L., Dixon L.K. (2018). Evaluation of protection induced by immunisation of domestic pigs with deletion mutant African swine fever virus benindeltamgf by different doses and routes. Vaccine.

[13] Reed L.J., Muench H. (1938). A simple method of estimating fifty percent endpoints. Am. J. Epidemiol..

[14] Borca M.V., Holinka L.G., Berggren K.A., Gladue D.P. (2018). Crispr-cas9, a tool to efficiently increase the development of recombinant African swine fever viruses. Sci. Rep..

[15] Carlson J., O’Donnell V., Alfano M., Velazquez Salinas L., Holinka L.G., Krug P.W., Gladue D.P., Higgs S., Borca M.V. (2016). Association of the host immune response with protection using a live attenuated African swine fever virus model. Viruses.

[16] Krug P.W., Holinka L.G., O’Donnell V., Reese B., Sanford B., Fernandez-Sainz I., Gladue D.P., Arzt J., Rodriguez L., Risatti G.R. (2015). The progressive adaptation of a Georgian isolate of African swine fever virus to vero cells leads to a gradual attenuation of virulence in swine corresponding to major modifications of the viral genome. J. Virol..

[17] Altschul S.F., Gish W., Miller W., Myers E.W., Lipman D.J. (1990). Basic local alignment search tool. J. Mol. Biol..

[18] O’Donnell V., Risatti G.R., Holinka L.G., Krug P.W., Carlson J., Velazquez-Salinas L., Azzinaro P.A., Gladue D.P., Borca M.V. (2017). Simultaneous deletion of the *9GL* and UK genes from the African swine fever virus Georgia 2007 isolate offers increased safety and protection against homologous challenge. J. Virol..

[19] Ruiz-Gonzalvo F., Carnero M.E., Bruyel V. (1981). Immunological Responses of Pigs to Partially Attenuated ASF and Their Resistance to Virulent Homologous and Heterologous Viruses.

[20] Sanford B., Holinka L.G., O’Donnell V., Krug P.W., Carlson J., Alfano M., Carrillo C., Wu P., Lowe A., Risatti G.R. (2016). Deletion of the thymidine kinase gene induces complete attenuation of the Georgia isolate of African swine fever virus. Virus Res..

[21] Neilan J.G., Zsak L., Lu Z., Kutish G.F., Afonso C.L., Rock D.L. (2002). Novel swine virulence determinant in the left variable region of the African swine fever virus genome. J. Virol..

